# The Contribution of Hypertensive Disorders of Pregnancy to Neonatal Unit Admissions and Iatrogenic Preterm Delivery at < 34^+0^ Weeks' Gestation in the UK: A Population‐Based Study Using the National Neonatal Research Database

**DOI:** 10.1111/1471-0528.17976

**Published:** 2024-10-06

**Authors:** Frances Conti‐Ramsden, Jessica Fleminger, Julia Lanoue, Lucy C. Chappell, Cheryl Battersby, the UK Neonatal Collaborative

**Affiliations:** ^1^ Department of Women and Children's Health King's College London London UK; ^2^ Neonatal Medicine, School of Public Health, Faculty of Medicine Imperial College London London UK; ^3^ Centre for Paediatrics and Child Health Imperial College London London UK

**Keywords:** ethnicity, foetal growth restriction, hypertension, neonatal unit, pregnancy, preterm birth

## Abstract

**Objectives:**

The objectives of this study were to (i) quantify the contribution of maternal hypertensive disorders of pregnancy (HDP) to iatrogenic preterm birth (PTB) and neonatal unit (NNU) admissions < 34^+0^ weeks and (ii) describe short‐term population‐level outcomes for HDP infants, exploring ethnic disparities and comparing outcomes by HDP exposure.

**Design:**

Retrospective population‐based study using the National Neonatal Research Database.

**Setting:**

England and Wales.

**Population:**

Infants born < 34^+0^ weeks and admitted to NNU 2012–2020.

**Methods:**

Descriptive statistics, linear and logistic regression models to compare outcomes between groups.

**Main Outcome Measures:**

Survival to discharge with/without comorbidity.

**Results:**

122 228 infants met inclusion criteria. Where collected, 49 839/114 164 (43.7%, 95% CI 43.4%–43.9%) of infants had an iatrogenic PTB. HDP was recorded in 16 510/122 228 (13.5%) of all infants and 13 560/49 839 (27.2%) of iatrogenic PTBs. HDP and/or foetal growth restriction (FGR) were recorded in 24 124/49 839 (48.4%) of iatrogenic PTBs. Singleton HDP infants < 10th BWC had ≥ 90% survival to discharge from 28 weeks' gestation, versus from 26 weeks' gestation for those born ≥ 10th BWC. In extreme preterm HDP infants (< 27 weeks), 27.3% of infants < 10th BWC died compared to 15.2% of those ≥ 10th BWC. Survival without comorbidity was ≥ 90% from 32 weeks' gestation in HDP infants across BWC.

**Conclusions:**

These contemporaneous population‐level data show that almost one in two PTB < 34^+0^ weeks' gestation are iatrogenic, with HDP and/or FGR being the major contributors to iatrogenic prematurity. This has substantial implications for strategies to reduce preterm birth in the UK and internationally. The data further inform antenatal and at‐birth counselling of HDP‐exposed infants.

## Abstract 2

This article includes Author Insights, a video abstract available at: https://vimeo.com/1004433858.

## Graphical abstract

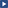

## 1 Introduction

Hypertensive disorders of pregnancy (HDP) are one of the most common complications of pregnancy, affecting approximately 10% of pregnant women, and are a leading cause of maternal morbidity and mortality globally [1]. The perinatal risks of HDP, primarily placental abruption, stillbirth, growth restriction, preterm birth and neonatal mortality, are well described [2, 3, 4, 5, 6], with the highest risk of adverse outcomes in women of Black ethnic backgrounds [6, 7, 8, 9]. Infant preterm birth confers lifelong increased risk of disability and chronic disease, with high healthcare costs that are inversely related to gestation at birth, as well as substantial impacts on the child's family [10, 11, 12, 13].

A recent UK study using maternal electronic health records has reported that just over one in two preterm births (births < 37 weeks' gestation) (52.8%) has an iatrogenic onset (or provider‐initiated onset) as opposed to a spontaneous onset [14], which is much higher than previously quoted UK figures (25%–30%) [15] and in keeping with rising rates of iatrogenic preterm birth (iPTB) reported internationally [16, 17]. The study also identified maternal HDP and foetal growth restriction (FGR) as two leading potential indications for iatrogenic preterm delivery alongside foetal distress and maternal diabetes [14]. If verified in other datasets, these findings have substantial implications for strategies to reduce preterm birth in the UK and internationally.

Comprehensive data on the contribution of HDP to iatrogenic prematurity at a population level and outcome data to inform joint obstetric neonatal decision‐making and antenatal counselling of women with HDP are lacking [18]. In the UK, almost all babies born less than 34^+0^ weeks' gestation (22–33^+6^ weeks) are admitted to neonatal units (NNUs) [18], and data are captured within the National Neonatal Research Database (NNRD) [20]. As such, the aims of this study were to (i) quantify the contribution of maternal HDP to iatrogenic preterm deliveries and admissions < 34^+0^ weeks' gestation at a population‐level and (ii) describe population‐level, short‐term neonatal outcomes to NNU discharge in infants born to mothers with HDP across birthweight centiles (BWCs), additionally comparing short‐term outcomes between HDP‐ and non‐HDP‐exposed infants and ethnic disparities in HDP infants.

## 2 Methods

We conducted a retrospective cohort study using prospectively collected data from the NNRD (Research Ethics Service approval [10/H0803/151]). The study was prospectively registered on ClinicalTrials.gov (NCT05015049) and received dedicated ethics approval (21/ES/0061). We report findings in line with RECORD guidelines [19]. Stakeholders were not involved in the design of this study. Data variables were limited to the data items held in the NNRD. Outcomes were defined to align with the neonatal core outcome set where possible [20].

### 2.1 Data Sources

The NNRD holds prospective, de‐identified, routinely recorded neonatal electronic health record data with complete coverage of infants admitted to National Health Service (NHS) NNUs in England and Wales since 2012 [18]. The high quality of neonatal data held in the NNRD has been confirmed by comparison with data from a randomised placebo‐controlled trial [18]. Freely available Office of National Statistics (ONS) England and Wales annual livebirth data, stratified by gestation and ethnicity, were used to generate population‐level denominator data [21].

### 2.2 Study Population

We included infants born between 1 January 2012 and 31 December 2020 at < 34^+0^ weeks' gestation admitted to an NHS NNU in England or Wales in this study. We performed data cleaning steps as reported in our previous study relating to late preterm and term infants [22], excluding infants with a confirmed congenital abnormality, those with implausible birthweight *Z*‐scores (≤ −4 or ≥ 4) and those with missing final discharge destination.

Infants were categorised into the following groups in keeping with British Association of Perinatal Medicine (BAPM) Guidelines [23]: (i) extreme preterm: born < 27^+0^ weeks' gestation; (ii) very preterm: born 27^+0^–31^+6^ weeks' gestation; and (iii) moderately preterm: born 32^+0^–33^+6^ weeks' gestation.

### 2.3 Exposure

To identify infants of mothers with HDP, maternal HDP diagnoses were extracted from antenatal, delivery and neonatal NNRD data items (Table S1). As delivery of infants to mothers with HDP < 34^+0^ weeks is more likely to have been associated with a clinical diagnosis of pre‐eclampsia (as opposed to gestational hypertension or chronic hypertension alone), all HDP codes were amalgamated into a single, binary HDP label.

### 2.4 Outcomes and Covariates

The primary outcome was infant survival to NNU discharge. Secondary outcomes were major comorbidities (retinopathy of prematurity, severe necrotising enterocolitis, severe brain injury and bronchopulmonary dysplasia). All outcome definitions are listed in Table S2.

Births were classified as having an iatrogenic onset of delivery if onset of labour was recorded as induction or ‘not in labour’ (i.e. infant delivered by caesarean section pre‐labour), consistent with provider‐initiated delivery. Similarly, caesarean sections were categorised according to whether they occurred pre‐labour, post‐induction or following spontaneous onset of labour. As all infants were delivered at < 34^+0^ weeks' gestation, all infants with an iatrogenic onset of delivery were classified as being cases of iatrogenic prematurity. Recorded potential indicators for iatrogenic premature birth were defined and summarised into the following categories: (i) HDP +/− FGR, (ii) antepartum haemorrhage, (iii) preterm +/− prolonged rupture of membranes and chorioamnionitis, (iv) multi‐foetal pregnancy, (v) other (including reduced foetal movements, rhesus and other haemolytic conditions, intrahepatic cholestasis of pregnancy, amniotic fluid and umbilical cord abnormalities).

Daily level of care was determined according to Health Resource Group definitions based on category of care received [24]. Birthweight *Z*‐scores were calculated with UK90 reference data using the LMS2z function of the SITAR package [25]. All outcome and diagnosis definitions and derived variables are listed in Table S2.

### 2.5 Statistical Methods

We report population proportions with 95% confidence intervals (CIs) calculated using the exact binomial test. Descriptive statistics were used to summarise survival and comorbidity outcomes in HDP‐exposed infants. We tested for differences in characteristics, outcomes and resource use between HDP‐exposed and non‐HDP infants using unadjusted and adjusted linear and logistic regression models for continuous and binary outcomes, respectively. Assumptions for linear models were checked by assessing the distribution of residuals. Variables associated with survival in HDP and all infants were tested using adjusted logistic regression models. Interactions were tested for using formal interaction terms in statistical models. To account for the large size of the dataset and multiple statistical hypothesis tests, a conservative, Bonferonni corrected *p*‐value threshold of *p* < 0.0005 was used to determine statistical significance of hypothesis tests (adjusted to account for 100 tests at a critical *p*‐value threshold of 0.05). Furthermore, given the large size of the dataset, small differences in outcomes between groups may be statistically significant without being of clinical significance. Therefore, results are presented as odds ratios with 95% CIs alongside *p*‐values to enable an appropriate interpretation of the findings in the context of clinical relevance. Importantly, for the primary regression models (outcome: survival to discharge), variables inputted into models other than HDP were selected a priori as those variables identified by expert clinicians as being the most important determinants of infant survival [23]. Missing data are reported in descriptive tables, with sensitivity analyses undertaken excluding variables with missingness > 5%. All data processing and analysis was performed in R version 4.3.1 (16 July 2023) [26].

## 3 Results

### 3.1 Contribution of Hypertensive Disorders of Pregnancy to NNU Admissions < 34^+0^ Weeks' Gestation

Of 133 798 reported livebirths at < 34^+0^ weeks' gestation in England and Wales 2012–2020 (Office of National Statistics data), 125 499 infants (93.8% of all livebirths, 95% CIs 93.7%–93.9%) had an NNRD record of NNU admission. Following exclusions, we included data for 122 288 infants who met study inclusion criteria (Figure S1). A maternal HDP was recorded in 16 510 of these 122 228 infants (13.5%, 95% CI 13.3%–13.7%). Onset of labour (spontaneous vs. iatrogenic onset) was collected in 114 164/122 288 (93.4%) of infants in the cohort overall. Where collected, 49 839/114 164 of infants in the cohort (43.7%, 95% CI 43.4%–43.9%) had an iatrogenic onset of delivery, defined as born following induction of labour or pre‐labour caesarean section. The proportion of infants admitted to NNU with a maternal HDP stratified by gestational age at delivery, year of birth and onset of labour is shown in Figure S2. Among infants exposed to maternal HDP (*n* = 16 510), 82.1% (95% CI 81.5%–82.7%) were iatrogenically premature (Figure S2), compared to 34.3% of non‐HDP infants.

### 3.2 Contribution of Hypertensive Disorders of Pregnancy to Iatrogenic Preterm Deliveries < 34^+0^ Weeks' Gestation

**FIGURE 1:**
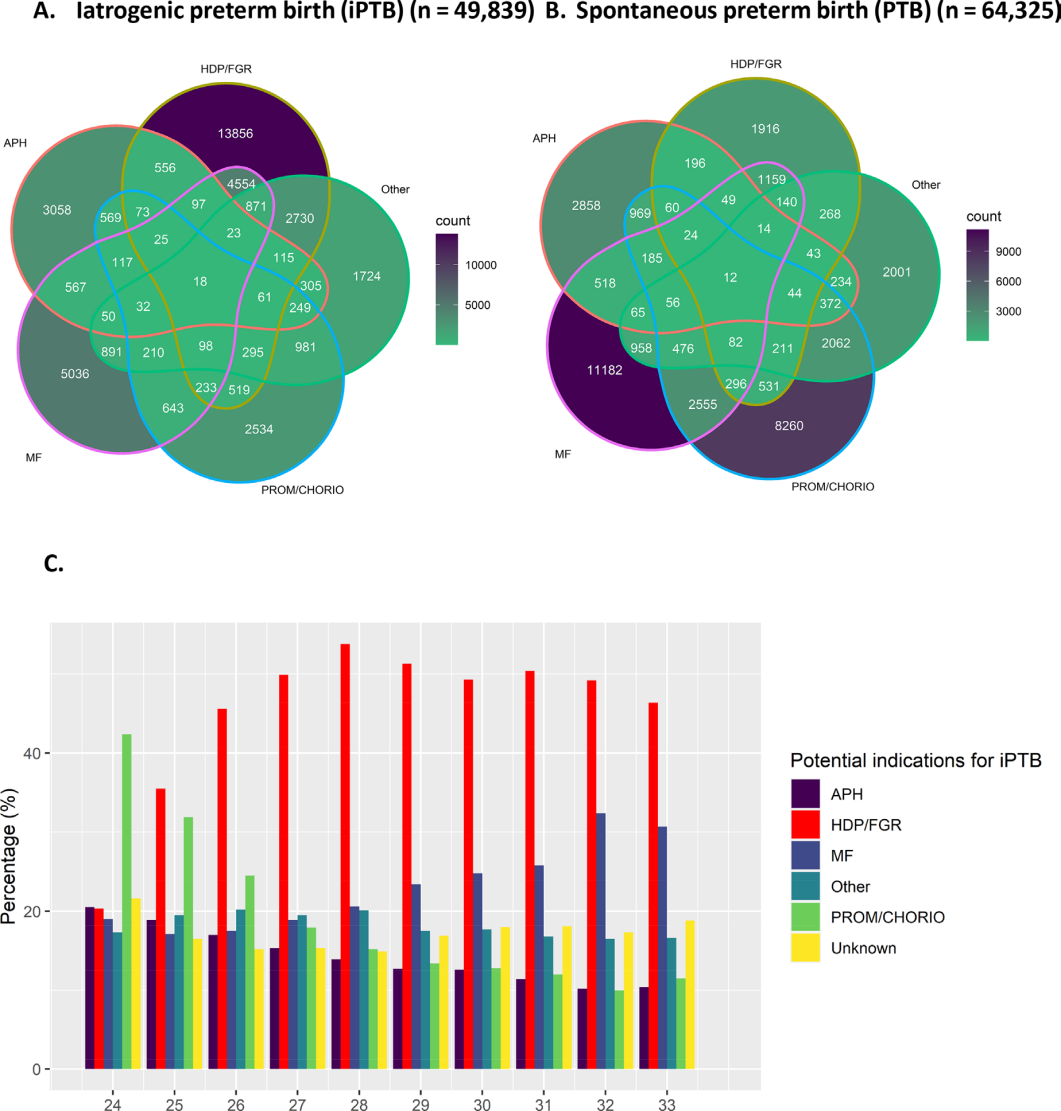
(A, B) Venn diagrams of potential indications/risk factors for preterm birth (PTB) in (A) iatrogenicPTB (iPTB), (B) spontaneous onset PTB, demonstrating HDP/FGR are the most common potential indicators for iPTB and multi‐foetal pregnancy is the most risk factor for spontaneous PTB. (C) Barplot of potential indications for iPTB (non‐mutually exclusive labels) in iatrogenically premature infants (*n* = 49 839) stratified by gestational age at delivery, demonstrating HDP/FGR is the most common potential indicator for iPTB from 25 week's gestation onwards. APH = antepartum haemorrhage; HDP/FGR = hypertensive disorder of pregnancy/foetal growth restriction; iPTB = iatrogenic preterm birth; MF = multi‐foetal pregnancy; PROM/CHORIO = prolonged or preterm rupture of membranes/chorioamnionitis.

Of 49 839 recorded iatrogenic preterm infants, over one in four had a maternal HDP (13 560/49 839 [27.2%, 95% CI 26.8%–27.6%]). The intersection of potential indications for iPTB in infants with iatrogenic versus spontaneous onset of delivery is shown in Figure 1A,B. HDP and/or FGR were the most common potential indications for iPTB, recorded in approximately one in two iatrogenic premature infants (24 124/49 839, 48.4%, 95% CI 48.0%–48.8%, Figure 1A). HDP and/or FGR were the most common recorded potential indications for iatrogenic preterm birth from 25 weeks' gestation in iatrogenically premature infants (Figure 1C). In iatrogenic premature infants with FGR (*n* = 20 808), one in three had a record of maternal HDP (6349/20 808, 30.5%). In infants with spontaneous onset of delivery, multi‐foetal pregnancy was the most common recorded potential indicator for premature delivery (17 771/64 325, 27.6%, 95% CI 27.3%–28.0%, Figure 1B).

### 3.3 Clinical Characteristics of Maternal HDP Versus Non‐HDP‐Exposed Infants

The majority (74.6%) of infants exposed to a maternal HDP were born by caesarean section compared to 44.9% of non‐HDP infants (Table S3). These were predominantly pre‐labour caesarean sections (Table S3), consistent with lower risk of prolonged/preterm rupture of membranes (OR 0.19, 95% CI 0.17–0.20, *p* < 0.0001) and chorioamnionitis (OR 0.21, 95% CI 0.17–0.25, *p* < 0.0001) in HDP‐ versus non‐HDP‐exposed infants (unadjusted analyses). HDP‐exposed infants were also more likely to have mothers who had received one or more doses of steroids than non‐HDP infants (15 361/16 510 (93.0%) HDP vs. 90 490/105 778 (85.5%) non‐HDP, Table S3). This remained significant after adjustment for gestational age at delivery, onset of labour and mode of delivery (aOR 2.00, 95% CI 1.78–2.24, *p* < 0.0001). In keeping with high risk of placental dysfunction in HDP, HDP‐exposed infants were more likely to be of low BWC than non‐HDP infants (Table S3).

### 3.4 Survival and Co‐Morbidity Outcomes for HDP‐Exposed Preterm Infants

**FIGURE 2:**
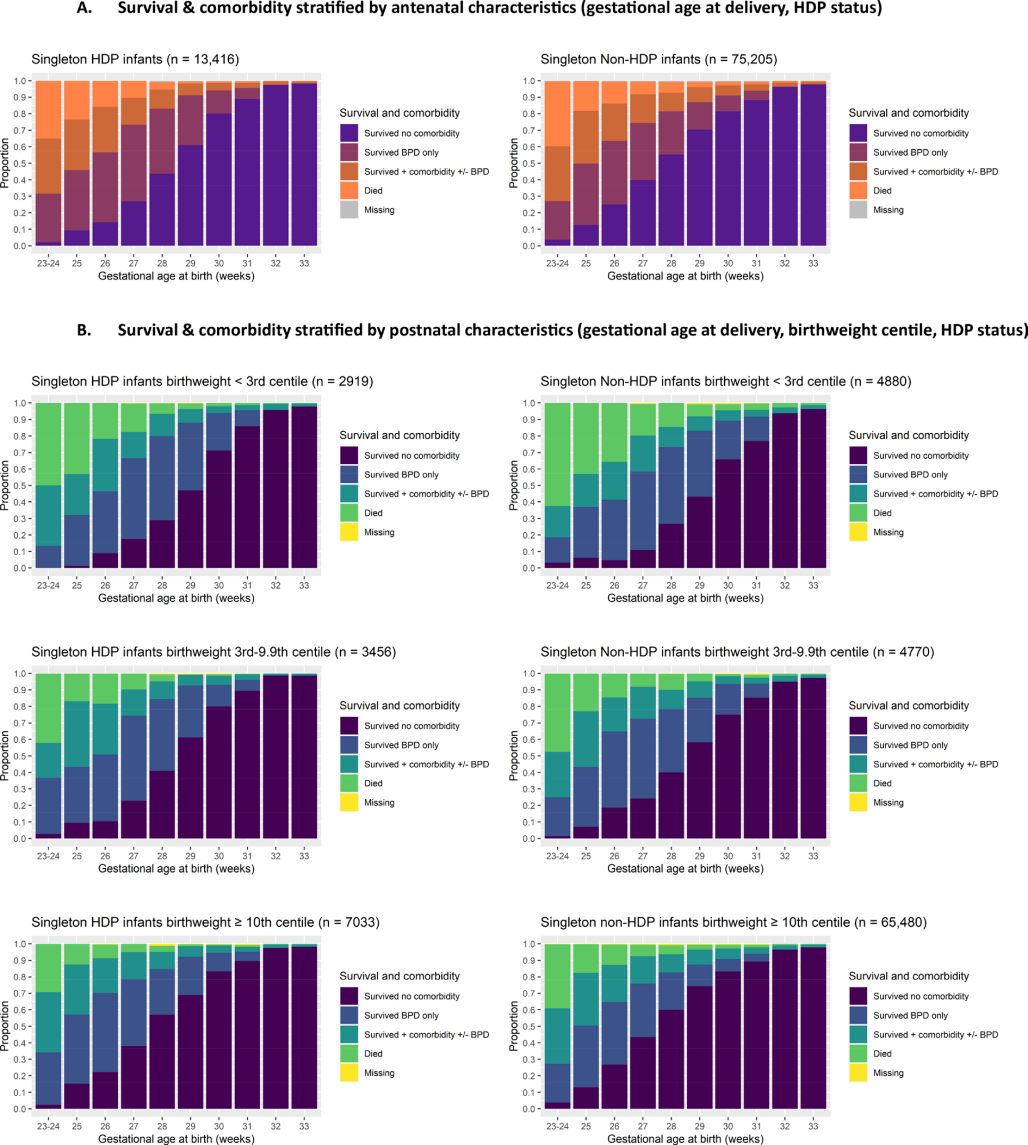
Survival with and without comorbidities stratified by (A) antenatal characteristics—gestational age at delivery and HDP status, (B) postnatal characteristics—gestational age at delivery, birthweight centile group and HDP status, in singleton infants from 23 week's gestation. Comorbidity = any of retinopathy of prematurity, severe necrotising enterocolitis or severe brain injury +/− bronchopulmonary dysplasia. BPD = bronchopulmonary dysplasia. Missing denotes infants with missing comorbidity data. For accompanying tabulated data, see Table S3.

Outcomes of infants exposed to a maternal HDP stratified by gestational age are summarised in Table 1. In a model including established clinical risk factors for survival [23], gestational age at birth, BWC and antenatal steroids were the primary drivers of HDP infant survival to discharge as demonstrated by independent association with the outcome, magnitude of odds ratios and highly significant *p*‐values (< 0.0001) (Table 2). As over 90% of HDP infants received antenatal steroids (see Section 3.3), survival to discharge stratified by gestational age (known antenatally), and gestational age and BWC (known postnatally) in HDP‐exposed singleton infants are illustrated in Figure 2 to inform antenatal counselling (singleton data—Table S4, multi‐foetal data—Table S5). Overall, survival to discharge was 78.1% (95% CI 75.4%–80.7%), 97.2% (95% CI 96.8%–97.6%) and 99.6% (95% CI 99.4%–99.8%) in extreme preterm, very preterm and moderately preterm singleton HDP‐exposed infants, respectively. In extreme preterm HDP infants overall, 27.3% of infants < 10th BWC died compared to 15.2% of those ≥ 10th BWC. Survival was 90% or more from 28 weeks' gestation in singleton HDP infants born < 10th centile and was 79.8% at 26 weeks' gestation. For singleton infants born at the 10th centile or more, survival was 90% or more from 26 weeks' gestation (Figure 2, Table S4).

**TABLE 1:** Neonatal outcomes, diagnoses and resource use in infants admitted to a neonatal unit at < 34 weeks' gestation in England and Wales 2012–2020 comparing those with and without a record of maternal hypertensive disorder of pregnancy (HDP) stratified by HDP type and gestational age at birth.

		Extreme preterm (< 27 weeks' gestation)		Very preterm (27–31^+6^ weeks' gestation)		Moderately preterm (≥ 32–33 + 6 weeks' gestation)	
		HDP, *N* = 1108	No HDP, *N* = 13 698	HDP, *N* = 7740	No HDP, *N* = 44 458	HDP, *N* = 7662	No HDP, *N* = 47 622
*Neonatal outcomes*							
Survival	Survived (all)	865 (78.07%)	10 091 (73.67%)	7521 (97.17%)	42 967 (96.65%)	7634 (99.63%)	47 294 (99.31%)
	Survived to discharge without major comorbidity	117 (10.56%)	1681 (12.27%)	5216 (67.39%)	32 282 (72.61%)	7516 (98.09%)	46 431 (97.50%)
	Survived to discharge with major comorbidity	746 (67.33%)	8366 (61.07%)	2273 (29.37%)	10 430 (23.46%)	113 (1.47%)	834 (1.75%)
	Missing comorbidity data^a^	2 (0.18%)	44 (0.32%)	32 (0.41%)	255 (0.57%)	5 (0.07%)	29 (0.06%)
*NNRD diagnoses*							
Major comorbidities	Any comorbidity (1 or more)	860 (77.62%)	10 196 (74.43%)	2388 (30.85%)	11 103 (24.97%)	126 (1.64%)	976 (2.05%)
	Treated ROP	194 (17.51%)	1834 (13.39%)	98 (1.27%)	369 (0.83%)	4 (0.05%)	11 (0.02%)
	BPD	737 (66.52%)	7837 (57.21%)	2033 (26.27%)	8431 (18.96%)	N/A	N/A
	Severe NEC	119 (10.74%)	1372 (10.02%)	167 (2.16%)	752 (1.69%)	18 (0.23%)	112 (0.24%)
	Severe brain injury	232 (20.94%)	3856 (28.15%)	428 (5.53%)	3169 (7.13%)	107 (1.40%)	867 (1.82%)
Growth restriction		403 (36.37%)	653 (4.77%)	3072 (39.69%)	5780 (13.00%)	2153 (28.10%)	6192 (13.00%)
Hypoglycaemia		391 (35.29%)	2486 (18.15%)	2392 (30.90%)	7632 (17.17%)	2194 (28.63%)	9242 (19.41%)
Seizures		43 (3.88%)	727 (5.31%)	85 (1.09%)	651 (1.46%)	40 (0.52%)	330 (0.69%)
Late onset sepsis		122 (11.01%)	1595 (11.64%)	207 (2.67%)	1024 (2.30%)	23 (0.30%)	156 (0.33%)
*Resource use*							
Total length of stay		100 [74–128]	94 [54–117]	50 [37–69]	46 [34–63]	22 [17–29]	20 [16–26]
Highest level of care	Intensive	1101 (99.37%)	13 579 (99.13%)	6604 (85.32%)	34 878 (78.45%)	2341 (30.55%)	12 596 (26.45%)
	High dependency	3 (0.27%)	32 (0.23%)	884 (11.42%)	7068 (15.90%)	2995 (39.09%)	17 476 (36.70%)
	Special	1 (0.09%)	17 (0.12%)	196 (2.53%)	2244 (5.05%)	2243 (29.27%)	17 091 (35.89%)
	Missing	3 (0.27%)	70 (0.51%)	56 (0.72%)	268 (0.60%)	83 (1.08%)	459 (0.96%)
Surfactant (any)		1036 (93.5%)	12 741 (93.01%)	4156 (53.70%)	22 076 (49.66%)	981 (12.80%)	6085 (12.78%)
Intubation (any)		957 (86.37%)	12 080 (88.19%)	2698 (34.86%)	15 702 (35.32%)	428 (5.59%)	2753 (5.78%)
Mechanical ventilation		1090 (98.38%)	13 438 (98.10%)	4798 (61.99%)	25 582 (57.54%)	1338 (17.46%)	8485 (17.82%)
Mechanical ventilation days		20 [7–37]	14 [5–31]	1 [0–4]	1 [0–3]	0 [0–0]	0 [0–0]
Non‐invasive ventilation		978 (88.27%)	11 541 (84.25%)	7302 (94.34%)	40 615 (91.36%)	4997 (65.22%)	29 163 (61.24%)
Non‐invasive ventilation days		71 [42–91]	65 [18–85]	13 [4–44]	9 [3–32]	1 [0–4]	1 [0–3]
Parenteral nutrition		1066 (96.21%)	12 662 (92.44%)	6625 (85.59%)	33 103 (74.46%)	2298 (29.99%)	9322 (19.57%)
Parenteral nutrition days		19 [12–33]	15 [9–26]	9 [6–14]	7 [1–11]	0 [0–4]	0 [0–0]
Blood products transfused		1052 (94.95%)	12 330 (90.01%)	3156 (40.78%)	15 384 (34.60%)	281 (3.67%)	2036 (4.28%)

*Note:* Data are summarised as counts (%) for categorical data, mean (standard deviation) for approximately normally distributed continuous variables or median [interquartile range] for skewed continuous variables.

Abbreviations: BPD: bronchopulmonary dysplasia; HD: high dependency care; IC: intensive care; NEC: necrotising enterocolitis; ROP: retinopathy of prematurity; SC: special care.

^a^ Missing comorbidity data primary due to missingness in bronchopulmonary dysplasia outcome variable, due to missing data in daily respiratory support data at 36 weeks (or day/penultimate day of discharge if discharged prior to 36 weeks) used for derivation (see Table S2 for details of comorbidity coding).

**TABLE 2:** Adjusted multivariable logistic regression model for binary outcome survival to discharge in HDP infants (complete case analysis, *N* = 12 963).

Variable	Odds ratio (95% confidence interval)	*p*
Gestational week at birth Reference: 23 weeks' gestation		
Gestational weeks = 24	2.08 (1.06–4.07)	0.0323
Gestational weeks = 25	4.12 (2.12–8)	< 0.00001
Gestational weeks = 26	6.44 (3.36–12.37)	< 0.00001
Gestational weeks = 27	11.5 (5.97–22.17)	< 0.00001
Gestational weeks = 28	22.28 (11.43–43.44)	< 0.00001
Gestational weeks = 29	83.53 (38.57–180.89)	< 0.00001
Gestational weeks = 30	128.01 (56.94–287.81)	< 0.00001
Gestational weeks = 31	125.41 (58.38–269.38)	< 0.00001
Gestational weeks = 32	290.79 (123.67–683.74)	< 0.00001
Gestational weeks = 33	519.19 (205.34–1312.78)	< 0.00001
Gestational days at birth, adjusted for gestational weeks Reference: +0 days		
Gestational days + 1	1.1 (0.75–1.62)	0.63559
Gestational days + 2	1.62 (1.07–2.45)	0.02158
Gestational days + 3	1.01 (0.7–1.47)	0.94831
Gestational days + 4	1.41 (0.95–2.11)	0.08914
Gestational days + 5	1.71 (1.16–2.53)	0.00719
Gestational days + 6	1.47 (1.01–2.15)	0.04387
Birthweight centile Reference: < 3rd centile		
Birthweight centile 3rd–9.9th	2.0 (1.52–2.63)	< 0.00001
Birthweight centile 10th–19.9th	3.36 (2.31–4.9)	< 0.00001
Birthweight centile 20th−29.9th	2.57 (1.66–3.98)	0.00002
Birthweight centile 30th−39.9th	2.73 (1.63–4.56)	0.00013
Birthweight centile 40th−49.9th	3.97 (1.98–7.95)	0.00001
Birthweight centile 50th−59.9th	3.49 (1.7–7.15)	0.00064
Birthweight centile 60th−69.9th	2.21 (0.99–4.91)	0.05146
Birthweight centile 70th−79.9th	1.4 (0.66–3)	0.38262
Birthweight centile 80th−89.9th	1.63 (0.64–4.16)	0.30929
Birthweight centile 90th+	0.97 (0.4–2.38)	0.95194
Maternal antenatal steroid course Reference: None given		
Complete course (2 or more doses)	3.4 (1.94–5.95)	0.00002
Incomplete course (1 dose)	3.59 (1.94–6.64)	0.00005
Mode of delivery Reference: Emergency caesarean section		
Spontaneous vaginal delivery	1.37 (0.91–2.05)	0.12823
Instrumental vaginal delivery	0.8 (0.25–2.59)	0.71355
Elective caesarean section	1.06 (0.24–4.72)	0.93778
Biological sex		
Female foetus	1.24 (1–1.54)	0.04928
Multi‐foetal pregnancy	0.75 (0.55–1.03)	0.07888

Abbreviation: HDP: hypertensive disorders of pregnancy.

Survival without comorbidity was 90% or more from 32 weeks' gestation in singleton HDP‐exposed infants across BWCs (Figure 2, Table S4). Bronchopulmonary dysplasia was the most common comorbidity in HDP‐exposed infants (Figure 2, Table 1), consistent with high rates of mechanical ventilation in HDP‐exposed infants, particularly at earlier gestations (Table 1).

### 3.5 Survival and Comorbidities in Infants Exposed to Maternal HDP Compared to Non‐HDP Infants

Figure 2 also illustrates survival and comorbidity in non‐HDP infants compared to HDP infants, showing overall lower survival in non‐HDP infants across gestational age and BWC groups. In survival models including all infants, gestational age at birth, BWC and antenatal steroids remained the primary drivers of infant survival to discharge. Interestingly, HDP exposure was also independently associated with increased survival (aOR 1.47, 95% CI 1.30–1.66, *p* < 0.0001, Table S6, complete case analysis, *N* = 95 993 infants analysed). Due to missingness > 5% in steroid administration and mode of delivery, a sensitivity analysis excluding these variables was completed yielding a similar result (aOR survival to discharge HDP vs. non‐HDP: aOR 1.53, 95% CI 1.37–1.70, *p* < 0.0001, *N* = 121 252 infants analysed). In subgroup analysis of infants with iatrogenic onset of delivery only (*N* = 49 839), the effect of HDP exposure on survival was very similar to that observed in the full cohort (aOR 1.46, 95% CI 1.26–1.70, *p* < 0.0001).

Whilst HDP‐exposed infants were more likely to survive than non‐HDP infants, a greater proportion of surviving infants born < 32 weeks survived with co‐morbidities, particularly treated retinopathy of prematurity and bronchopulmonary dysplasia, than non‐HDP infants (Table 1, Figure 2).

### 3.6 Ethnic Disparities in Infants Exposed to Maternal HDP

HDP‐exposed infants were disproportionately born to women of Black and Asian ethnic backgrounds in multivariable models adjusted for maternal age, parity, foetus number, index of multiple deprivation and medical comorbidities (aOR HDP infant Black vs. White: 2.20, 95% CI 2.08–2.35, *p* < 0.0001; aOR HDP infant Asian vs. White: 1.34, 95% CI 1.29–1.43, *p* < 0.0001). The number and proportion of infants born to women of minority ethnic backgrounds was similar to those reported by ONS national England and Wales statistics 2014–2020 (Table S7).

Cumulative frequency graphs of gestational age at delivery stratified by maternal ethnic group (Asian, Black and White) in HDP‐exposed and all infants is shown in Figure S3. A cumulative frequency of 50% was achieved at 31^+1^ weeks of gestation in HDP‐exposed infants born to mothers of Black ethnic backgrounds compared to 31^+6^ weeks of gestation in HDP‐exposed infants born to mothers of White ethnic backgrounds (−5 days). HDP‐exposed infants born to mothers of Black ethnic backgrounds had a higher likelihood of being born extremely or very preterm in models adjusted for maternal age, parity, multi‐foetal pregnancy, index of multiple deprivation and comorbidities (extreme preterm birth Black vs. White maternal ethnicity in HDP‐exposed infants: aOR 1.41, 95% CI 1.16–1.72, *p* = 0.0004; very preterm birth Black vs. White maternal ethnicity in HDP‐exposed infants: aOR 1.26, 95% CI 1.13–1.40, *p* < 0.0001). However, Black and Asian maternal ethnicity were also strongly associated with extreme preterm birth in all infants (extreme preterm birth Black vs. White all infants: aOR 2.07, 95% CI 1.94–2.21, *p* < 0.0001; extreme preterm birth Asian vs. White all infants: aOR 1.31, 95% CI 1.23–1.39, *p* < 0.0001). In models adjusted for gestational age at delivery, there was no association between maternal ethnicity and infant survival to NNU discharge.

## 4 Discussion

### 4.1 Summary of Main Findings

Our study demonstrates maternal HDP and associated placental disorders are a substantial contributor to national UK infant NNU admissions and iatrogenic preterm births < 34^+0^ weeks'. Over one in ten (13.5%) infant NNU admissions were associated with a maternal HDP. Over four in ten (43.7%) infants had an iatrogenic onset of delivery; maternal HDP and/or FGR were the most commonly recorded potential indications, with one in two iatrogenically premature infants exposed to HDP and/or FGR and one in four infants exposed to HDP alone.

We have demonstrated the critical importance of gestational age, steroid administration and BWC in determining HDP infant survival. To our knowledge this is the first study to report UK population‐level outcome data for infants of maternal HDP, stratified by gestational age and BWCs, that can be used at antenatal and post‐birth time points for joint obstetric and neonatal decision‐marking regarding timing of birth and antenatal counselling in HDP. For example, singleton HDP infants with birthweight ≥ 10th centile have > 90% survival from 26 weeks' gestation. However, small‐for‐gestational‐age HDP infants (BWC < 10th centile) have ~80% survival at 26 weeks and > 90% survival at 28 weeks' gestation.

This is also the first study to report UK population‐level data on ethnic disparities in HDP. In the UK, the incidence of HDP exposure in infants delivered < 34 weeks varies substantially by maternal ethnicity with infants born to Black women having more than twice the risk of exposure to a HDP than infants born to women of White ethnic backgrounds in adjusted analyses, as well as earlier gestational age of delivery in Black and Asian HDP‐exposed infants.

### 4.2 Strengths and Limitations

The strengths of this study include population‐level UK data, as this and other studies have shown the NNRD captures almost all babies delivered < 34^+0^ weeks across the UK [18]. Furthermore, missingness in variables included in regression models was generally very low, and sensitivity analysis excluding variables with missingness > 5% did not substantially alter results. Importantly, this study cannot comment on infants who die prior to admission to a NNU, such as stillbirths and intrapartum deaths, as they will not have an NNU record. The characteristics of HDP‐exposed infants < 34^+0^ weeks in the cohort is in keeping with anticipated clinical phenotypes [1, 27], providing further assurance of HDP coding in the NNRD [22]. The granularity and scope of data available in the NNRD (including maternal comorbidities) enabled us to identify potential indications for iatrogenic delivery as well as adjust for relevant confounders in regression models, including all major risk factors for survival as identified in the BAPM framework for extreme preterm birth [23]. However, maternal body mass index is not available in the NNRD, and may be a potential mediator of higher risk of HDP and earlier preterm birth in women of ethnic minority groups, as shown in the study by Aughey et al. [14] Whilst this study highlights the utility of the NNRD for research at the intersection of obstetrics and neonatology, there is further opportunity to maximise its utility through linkage to maternal datasets with variables including BMI. The full potential of these datasets will be realised through the creation of longitudinal maternal‐neonatal electronic health records, which will be a valuable resource for research.

### 4.3 Interpretation in the Light of Other Studies

Our population estimate of 43.7% of UK preterm births < 34^+0^ weeks' gestation being iatrogenic‐onset (excluding infants with recorded congenital abnormalities) is much higher than the regularly quoted figure of one in four preterm births being iatrogenic [28], and is supported by the figure of 47.7% reported by a recent, smaller English maternal electronic health record cohort study conducted over a shorter timescale (2015–2017 data) [14]. The same cohort study identified FGR (22.9%) and maternal HDP (18.2%) as the most commonly recorded potential indicators for iatrogenic preterm birth < 37 weeks alongside suspected foetal distress (26.8%) [14]. Our larger, UK population‐level study further reports that in infants born < 34^+0^ weeks' (the population which is admitted to NNU and therefore has greatest cost to the NHS, the infant and their family) [10], HDP and FGR are the primary drivers of iatrogenic birth, with one or both being recorded in approximately 50% of cases. Both these findings are highly relevant to UK preterm birth prevention strategies.

Our models also provide population‐level evidence to corroborate the variables included in the BAPM framework for extreme preterm birth, affirming that BWC, antenatal steroids and foetal sex are independently associated with HDP and non‐HDP infant survival alongside gestational age, and further highlighting BWC as the second most influential variable affecting survival after gestational age (Table 2) [23]. However, multi‐foetal pregnancy, which is currently included as a risk factor in the BAPM framework, was not independently associated with survival in our models. Notably, in keeping with high rates of iatrogenic delivery of HDP‐exposed infants (approximately 80% in this study), it is reassuring that > 90% of HDP‐exposed infants received one or more doses of steroids. This is higher than in a recently reported cohort study (where 80% of HDP infants delivered < 35 and < 32 weeks received steroids) [29], though the exact timing of steroid administration with respect to delivery is not available in the NNRD.

HDP exposure, independently of gestational age, BWC, steroid administration, foetal sex, plurality and mode of delivery, was associated with higher odds of survival (1.5×) compared to non‐HDP‐exposed infants, with an odds ratio equivalent to the effect of +3 days gestational age. A 2017 systematic review and meta‐analysis of preterm infants (< 37 weeks' gestation) born to mothers with a HDP also reported a pooled adjusted OR for survival of 1.5 (95% CI 1.2–1.9, 3 studies) compared to non‐HDP infants [30]. However, these findings need to be interpreted with caution, as non‐HDP preterm infants do not represent a healthy control group, and their poorer outcomes maybe multifactorial. The non‐HDP preterm infant group is typified by higher rates of exposure to labour, spontaneous vaginal delivery and infective complications such as ruptured membranes and chorioamnionitis, as evident in our data. Furthermore, they may be less likely to undergo regular antenatal surveillance than HDP‐exposed infants who are more likely to undergo regular foetal ultrasound scans. As such, it may be prudent to interpret the data as preterm birth overall carrying a high risk of adverse infant outcomes, with infants born following spontaneous labour and/or infective processes having worse short‐term outcomes than iatrogenically delivered HDP‐exposed infants. However, further exploration of the hypothesis that maternal hypertension is an adaptive response to optimise survival of a stressed (hypoxic) foetus, leading to accelerated organ maturation [31], as well as investigation of glucocorticoid regulation in stressed foetuses [32], with concomitant impacts on long‐term health, is needed.

Investigation of the relationship between maternal ethnicity and HDP is ongoing [7, 33]. Notably, whilst the risk of extreme and very premature birth was highest in HDP‐exposed infants born to women of Black and Asian versus White ethnic backgrounds, adjusted survival did not vary by maternal ethnicity. Therefore, efforts to tackle ethnic disparities in HDP‐exposed infants must focus on the maternal disease, particularly prediction and prevention. Interestingly, higher risk of earlier preterm birth in women of ethnic minority backgrounds than in white women (irrespective of HDP) has been previously reported in a large, single‐centre study (data 1988–1998) [34].

## 5 Conclusion

This UK population‐level study demonstrates that a high proportion (approximately 45%) of preterm births < 34^+0^ weeks are iatrogenic‐onset, with one in two of these iatrogenic preterm births likely being associated with HDP and FGR. Therefore, clinicians and policy‐makers addressing premature birth in maternity must recognise and address the burden of iatrogenic (primarily HDP and associated placental disorders) in addition to spontaneous drivers of preterm birth [35]. To reduce the neonatal burden of iatrogenic preterm births, our findings provide justification to focus resources and research efforts on prevention of HDP, disease‐modifying treatment of HDP (rather than reliance on delivery of the placenta) and optimisation of foetal blood flow and growth in placental disorders. We also provide real‐world, population‐level data to inform antenatal and post‐birth counselling of HDP‐exposed infants, highlighting the critical importance of gestational age at birth and BWC in driving survival in HDP‐exposed infants < 34^+0^ weeks' gestation.

## Author Contributions

F.C.‐R., L.C.C. and C.B. conceived the study. J.L. accessed the NNRD database and performed data extraction. J.L. and J.F. performed data cleaning. F.C.‐R. carried out data analysis. F.C.‐R. drafted the first version of the manuscript which was edited and approved by all the other authors.

## Conflicts of Interest

C.B. is funded by the UK NIHR through an Advanced Fellowship Award, has received support from Chiesi Pharmaceuticals to attend educational conferences and has been an investigator on research grants from the National Institute of Health Research. C.B. is deputy chair for the NIHR Prioritisation committee for Hospital‐based care. F.C.‐R., J.F., J.L. and L.C.C. report no conflicts of interest.

## Supporting information


Data S1.

Video S1.

## Data Availability

Data are available upon reasonable request. Applications to use the data used within this project should be made to the Neonatal Data Analysis Unit, Imperial College London.
