# Supplementary material for: The Contribution of Hypertensive Disorders of Pregnancy to Neonatal Unit Admissions and Iatrogenic Preterm Delivery at < 34+0 Weeks' Gestation in the UK: A Population‐Based Study Using the National Neonatal Research Database
Source: BJOG. 2024 Oct 6;132(3):306–17. doi: 10.1111/1471-0528.17976 (PMC11704029; doi:10.1111/1471-0528.17976)

**Supplementary Tables**

**Table S1.** NNRD extraction procedures for hypertensive disorder of pregnancy diagnosis.

| **Variable** | **NNRD extraction procedure** |
| --- | --- |
| Hypertensive disorder of pregnancy | *Antenatal variables*  Variable: *ProblemsMedicalMother*  Coding: Dichotomous, code 12 (chronic hypertension) = TRUE, other codes = FALSE. |
|  | *Antenatal variables*  Variable: *ProblemsDuringPregnancy*  Coding: Dichotomous, code 30 (Pregnancy induced hypertension) = TRUE, other codes = FALSE. |
|  | *Antenatal variables*  Variable: *ProblemsDuringPregnancy*  Coding: Dichotomous, code 31 (Pre-eclampsia) = TRUE  AND/OR  *Discharge diagnosis table*  Coding: TRUE if any of the following codes:  15389 (mild preeclampsia)  15390 (moderate preeclampsia)  15391 (severe preeclampsia) |
|  | *Antenatal variables*  Variable: *ProblemsDuringPregnancy*  Coding: Dichotomous, code 32 (maternal HELLP) = TRUE, other codes = FALSE. |
|  | *Labour and delivery*  Variable: *Drugs in Labour*  Coding: Dichotomous, code 18 (antihypertensive) = 1, other codes = 0. |

**Table S2.** NNRD extraction procedures and definition of derived outcomes, risk factors and covariates.

| **Variable** | **NNRD extraction procedure** |
| --- | --- |
| Survival to discharge from neonatal unit | WHERE  Discharge destination at final admission = ‘Died’  THEN (0)  0 = Died  1 = Survived to discharge  9 = Unknown |
| Survival to discharge from neonatal care without comorbidity | WHERE  Survival == 1 AND  IF Treated_ROP = 0 AND BPD = 0 AND  SevereNEC = 0 AND Brain injury = 0  THEN  0 = Died  1 = Survival with comorbidities  2 = Survival no comorbidities |
| Fetal growth restriction *(Risk factor for indicated preterm birth)* | *Antenatal variables*  Variable: *ProblemsDuringPregnancy*  Coding: TRUE if any of the following codes:  "Intrauterine growth restriction" (10)  "Poor biophysical profile" (11)  AND/OR  *Discharge diagnosis table*  Variables: *PrincipalDiagnosisAtDischarge* and text in diagnosis table have any of the following terms (== TRUE):  '15510': 'INTRAUTERINE GROWTH RESTRICTION (IUGR)'  '1010152': 'REVERSED DIASTOLIC FLOW (MATERNAL DOPPLERS)'  '15505': 'SMALL FOR DATES - NO MALNUTRITION (SGA/IUGR)'  '1010151': 'ABSENT DIASTOLIC FLOW (MATERNAL DOPPLERS)'  '15509': 'FETAL GROWTH RETARDATION'  '1010295': 'SMALL FOR GESTATIONAL AGE'  AND/OR  Birthweight centile (UK90) < 3^rd^ centile |
| Antepartum haemorrhage *(Risk factor for indicated preterm birth)* | *Antenatal variables*  Variable: *ProblemsDuringPregnancy*  Coding: TRUE if any of the following codes:  27 - Placental abruption  28 - Placenta praevia  29 - Other antepartum haemorrhage |
| Rupture of membranes and chorioamnionitis *(Risk factor for indicated preterm birth)* | *Antenatal variables*  Variable: *ProblemsDuringPregnancy*  Coding: TRUE if any of the following codes:  18 - Chorioamnionitis  19 - Preterm rupture of membranes  20 - Prolonged rupture membranes  21 - Cervical suture  22 - Maternal Gp B Strep |
| Multi-fetal pregnancy *(Risk factor for indicated preterm birth)* | *Antenatal variables*  Fetus number > 1  OR  If fetus number missing, use:  *Antenatal variables*  Variable: *ProblemsDuringPregnancy*  Coding: TRUE if any of the following codes:  17 - Twin to twin transfusion |
| Other risk factor for indicated preterm birth | *Antenatal variables*  Variable: *ProblemsDuringPregnancy*  Coding: TRUE if any of the following codes:  12 - Reduced fetal movements  13 - Oligohydramnios  14 - Polyhydramnios  16 - Cord problems  24 - Other infection  25 - Rhesus  26 - Other haemolytic disease  34 - Cholestasis of pregnancy |
| Hypoglycaemia diagnosis | The following terms or codes in discharge diagnoses or  the diagnosis table:  '15773': 'HYPERINSULINAEMIC HYPOGLYCAEMIA'  '15771': 'IATROGENIC NEONATAL HYPOGLYCAEMIA'/'HYPOGLYCAEMIA'/'NEONATAL HYPOGLYCAEMIA'/'OTHER NEONATAL HYPOGLYCAEMIA'  '500530': 'DRUG INDUCED HYPOGLYCAEMIA' |
| Brain injury (UK Department of Health Definition) | For published definitions see:  Gale C, Statnikov Y, Jawad S On behalf of the Brain Injuries expert working group*, et al.* Neonatal brain injuries in England: population-based incidence derived from routinely recorded clinical data held in the National Neonatal Research Database. *Archives of Disease in Childhood - Fetal and Neonatal Edition*2018;**103:**F301-F306.   - Gale C, Ougham K, Jawad S, Uthaya S, Modi N. Brain injury occurring during or soon after birth: annual incidence and rates of brain injuries to monitor progress against the national maternity ambition 2018 and 2019 national data. *29*. 2021. doi:10.25561/87336   In preterm babies where:  ‘HIE’ = 1 OR  ‘IVH’ =1 OR  ‘PerinatalStroke’ =1 OR  ‘Kernicterus’ = 1 OR  ‘CNSInfection’ =1 OR  CPVL = 1 OR  Seizures = 1  THEN (1)  *Individual definitions:*  HIE: Defined as diagnoses of ‘Grade 3’ or ‘Grade 2’ HIE from all diagnosis fields in the NNRD  OR  Therapeutic Hypothermia for 2 or more consecutive days  IVH: Defined as diagnosis of ‘Grade ‘3’ or ‘Grade 4’ IVH from Cranial scans table in the NNRD  OR  Any of the following diagnoses in any of the diagnosis fields in the NNRD:   - SUBDURAL HAEMORRHAGE (DUE TO BIRTH INJURY) - CEREBRAL HAEMORRHAGE DUE TO BIRTH INJURY - TRAUMATIC INTRAVENTRICULAR HAEMORRHAGE - SUBARACHNOID HAEMORRHAGE - SUBARACHNOID HAEMORRHAGE DUE TO BIRTH INJURY - TENTORIAL TEAR DUE TO BIRTH INJURY - INTRACRANIAL LACERATION AND HAEMORRHAGE DUE TO BIRTH INJURY - INTRACRANIAL HAEMORRHAGE (UNKNOWN OR UNSPECIFIED CAUSE) - INTRACEREBRAL HAEMORRHAGE (TERM INFANT) - INTRAVENTRICULAR HAEMORRHAGE (PERINATAL) - POST-HAEMORRHAGIC HYDROCEPHALUS   OR  Any of the following procedures in any of the procedures fields in the NNRD:   - VENTRICULOPERITONEAL OR OTHER VENTRICULAR SHUNT - EXTERNAL VENTRICULAR DRAIN - VENTRICULAR DRAIN WITH RESERVOIR - INSERTION OF VENTRICULAR PERITONEAL SHUNT - INSERTION OF RICKHAM RESERVOIR - INSERTION OF VENTRICULO-ATRIAL CSF SHUNT - INSERTION OF VENTRICULO-PERITONEAL CSF SHUNT - CREATION OF VENTRICULOPERITONEAL SHUNT   PerinatalStroke: Defined as any of the following diagnoses in any of the diagnosis fields within the NNRD:   - NEONATAL STROKE - INFARCTION: MIDDLE CEREBRAL ARTERY - CEREBROVASCULAR ACCIDENT - CEREBRAL VENOUS THROMBOSIS - NEONATAL CEREBRAL ISCHAEMIA   Kernicterus:  Defined as any of the following diagnoses in any of the diagnosis fields within the NNRD:   - KERNICTERUS - BILIRUBIN ENCEPHALOPATHY   Seizures: Defined as any entry of ‘Convulsions’ in the Daily summary table in the NNRD  CNSinfection: Defined as any of the following diagnoses in any diagnosis field within the NNRD:   - ENCEPHALITIS - CANDIDA ENCEPHALITIS - BACTERIAL MENINGITIS - VIRAL MENINGITIS - MENINGITIS - BACTERIAL (SPECIFIC ORGANISM)   Specific options found in NNRD:   - - MENINGITIS – STREPTOCOCCAL   - MENINGITIS – CANDIDA - MENINGITIS - BACTERIAL (UNKNOWN OR UNSPECIFIED CAUSE) - CONGENITAL HERPES   OR  Any entry of any growth from a CSF culture  CPVL: Defined as being born at less than 37 weeks gestational age AND having any of the following diagnoses in any of the diagnosis field in the NNRD:   - CYSTIC PERIVENTRICULAR LEUKOMALACIA   OR  Being born < 37 weeks gestational age and having a diagnosis of PVL from Cranial scans table in the NNRD |
| Treated retinopathy of prematurity | WHERE  Surgery for ROP listed OR  Cryotherapy, laser therapy, anti-VEGF therapy, other treatment listed in right or left eye  OR  Avastin given  AND  GestationWeeks at birth < 32  AND  FirstBirthWeight < 1500g  THEN == 1, ELSE == 0 |
| Bronchopulmonary dysplasia | WHERE  GestationWeeks at birth < 32 weeks  AND  Any respiratory support given at WEEK 36 corrected gestational age  THEN  0 = Died before week 36  1 = No respiratory support given at 36 weeks  2 = Respiratory support at 36 weeks  9 = Unknown  * Where no data for week 36 available due to early discharge, data is taken from the final or penultimate day on the unit |
| Severe necrotising enterocolitis | Necrotising enterocolitis resulting in death or requiring surgery  0 = No severe NEC  1 = Severe NEC present  9 = Unknown |
| Growth velocity | WeightAtDischarge MINUS FirstBirthWeight  DIVIDED BY  Total length of stay in neonatal unit |
| Total length of stay in neonatal unit | SUM (Discharge time – Admission time) for each episode of care  In days |
| Number of days of intensive/high dependency/special care | SUM DAILY RECORDS WHERE  ‘HRG2016’ = intensive care / high dependency / special care as appropriate |

**Table S3.** Baseline, antenatal and delivery characteristics of infants admitted to a neonatal unit at < 34 weeks’ gestation in England and Wales 2012-2020 comparing those with and without a record of maternal hypertensive disorder of pregnancy (HDP) stratified by HDP status and gestational age at birth.

|  | | **Extreme preterm**  **(< 27 weeks’ gestation)** | | **Very preterm**  **(27-31^+6^ weeks’ gestation)** | | **Moderately preterm**  **(32-33^+6^ weeks’ gestation)** | |
| --- | --- | --- | --- | --- | --- | --- | --- |
|  | | **HDP**  **N = 1108** | **No HDP**  **N = 13,698** | **HDP**  **N = 7740** | **No HDP**  **N = 44,458** | **HDP**  **N = 7662** | **No HDP**  **N = 47,622** |
| **Baseline characteristics** | | | | | | | |
| Sex | Female | 576 (51.99%) | 6255 (45.66%) | 3941 (50.92%) | 19,548 (43.97%) | 3888 (50.74%) | 21,708 (45.58%) |
|  | Male | 532 (48.01%) | 7442 (54.33%) | 3792 (48.99%) | 24,886 (55.98%) | 3770 (49.20%) | 25,891 (54.37%) |
|  | *Missing* | 0 (0.00%) | 1 (0.01%) | 7 (0.09%) | 24 (0.05%) | 4 (0.05%) | 23 (0.05%) |
| Birthweight (g) |  | 650 [556-765] | 754 [640-870] | 1140 [930-1356] | 1360 [1130-1580] | 1675 [1476-1910] | 1915 [1700-2125] |
|  | *Missing* | 0 (0.00%) | 26 (0.19%) | 7 (0.09%) | 48 (0.11%) | 5 (0.07%) | 29 (0.06%) |
| Birthweight centile |  | 18.1 (21.04) | 39.5 (21.61) | 19.1 (22.06) | 42.4 (26.91) | 25.6 (25.82) | 43.0 (28.07) |
| Birthweight centile <10th |  | 576 (51.99%) | 1104 (8.06%) | 3807 (49.19%) | 6820 (15.34%) | 2923 (38.15%) | 7369 (15.47%) |
| Number of fetuses | Singleton | 972 (87.73%) | 10,380 (75.78%) | 6676 (86.25%) | 31,626 (71.14%) | 5773 (75.35%) | 33,309 (69.94%) |
|  | Multi-fetal | 136 (12.27%) | 3317 (24.22%) | 1064 (13.75%) | 12,832 (28.86%) | 1889 (24.65%) | 14,310 (30.05%) |
|  | *Missing* | 0 (0.00%) | 1 (0.01%) | 0 (0.00%) | 0 (0.00%) | 0 (0.00%) | 3 (0.01%) |
| Maternal age (years) |  | 31 [27-36] | 31 [26-35] | 32 [27-36] | 31 [26-35] | 32 [27-36] | 31 [26-35] |
|  | *Missing* | 3 (0.27%) | 75 (0.55%) | 47 (0.61%) | 318 (0.72%) | 45 (0.59%) | 462 (0.97%) |
| Maternal parity | Primiparous | 466 (42.06%) | 4403 (32.14%) | 3285 (42.44%) | 13,884 (31.23%) | 3237 (42.25%) | 14,790 (31.06%) |
|  | *Missing* | 74 (6.68%) | 1242 (9.07%) | 504 (6.51%) | 4650 (10.46%) | 593 (7.74%) | 5368 (11.27%) |
| Ethnicity (maternal) | Asian | 147 (13.27%) | 1694 (12.37%) | 1140 (14.73%) | 4658 (10.48%) | 1026 (13.39%) | 4531 (9.51%) |
|  | Black | 190 (17.15%) | 1408 (10.28%) | 1017 (13.14%) | 2723 (6.12%) | 745 (9.72%) | 2153 (4.52%) |
|  | Mixed | 20 (1.81%) | 235 (1.72%) | 109 (1.41%) | 695 (1.56%) | 110 (1.44%) | 674 (1.42%) |
|  | Other | 16 (1.44%) | 298 (2.18%) | 136 (1.76%) | 795 (1.79%) | 147 (1.92%) | 880 (1.85%) |
|  | White | 589 (53.16%) | 8126 (59.32%) | 4252 (54.94%) | 28,566 (64.25%) | 4504 (58.78%) | 31,340 (65.81%) |
|  | *Missing* | 146 (13.18%) | 1937 (14.14%) | 1086 (14.03%) | 7021 (15.79%) | 1130 (14.75%) | 8044 (16.89%) |
| Most deprived IMD quintile (maternal) |  | 358 (32.31%) | 4576 (33.41%) | 2333 (30.14%) | 13,786 (31.01%) | 2073 (27.06%) | 13,791 (28.96%) |
|  | *Missing* | 29 (2.62%) | 309 (2.26%) | 152 (1.96%) | 1127 (2.53%) | 178 (2.32%) | 1353 (2.84%) |
| **Antenatal characteristics** | | | | | | | |
| Medical history (maternal) | Diabetes | 36 (3.25%) | 174 (1.27%) | 364 (4.70%) | 892 (2.01%) | 439 (5.73%) | 1299 (2.73%) |
|  | SLE | 7 (0.63%) | 15 (0.11%) | 41 (0.53%) | 68 (0.15%) | 32 (0.42%) | 90 (0.19%) |
|  | Renal failure | 13 (1.17%) | 17 (0.12%) | 89 (1.15%) | 57 (0.13%) | 62 (0.81%) | 54 (0.11%) |
|  | Renal transplant | 3 (0.27%) | 2 (0.01%) | 31 (0.40%) | 21 (0.05%) | 24 (0.31%) | 27 (0.06%) |
| Obstetric complications | Fetal growth restriction | 275 (24.82%) | 340 (2.48%) | 2129 (27.51%) | 3868 (8.70%) | 1382 (18.04%) | 4381 (9.20%) |
|  | Placental abruption | 21 (1.90%) | 438 (3.20%) | 120 (1.55%) | 1343 (3.02%) | 89 (1.16%) | 945 (1.98%) |
|  | Gestational diabetes | 45 (4.06%) | 238 (1.74%) | 488 (6.30%) | 2011 (4.52%) | 704 (9.19%) | 2693 (5.65%) |
| Antenatal corticosteroids | Complete | 870 (78.52%) | 8830 (64.46%) | 6319 (81.64%) | 30,443 (68.48%) | 6248 (81.55%) | 31,573 (66.30%) |
|  | Incomplete | 170 (15.34%) | 2905 (21.21%) | 981 (12.67%) | 8303 (18.68%) | 773 (10.09%) | 8436 (17.71%) |
|  | None given | 17 (1.53%) | 850 (6.21%) | 151 (1.95%) | 2311 (5.20%) | 229 (2.99%) | 3127 (6.57%) |
|  | *Missing* | 51 (4.60%) | 1113 (8.13%) | 289 (3.73%) | 3401 (7.65%) | 412 (5.38%) | 4486 (9.42%) |
| Antenatal magnesium sulfate | Given | 655 (59.12%) | 6205 (45.30%) | 3870 (50.00%) | 14272 (32.10%) | 2220 (28.97%) | 5395 (11.33%) |
|  | Not given | 204 (18.41%) | 3842 (28.06%) | 1546 (19.97%) | 14,786 (33.26%) | 2386 (31.14%) | 20,839 (43.76%) |
|  | *Missing* | 249 (22.47%) | 3650 (26.65%) | 2324 (30.03%) | 15,400 (34.64%) | 3056 (39.89%) | 21,388 (44.91%) |
| **Delivery characteristics** | | | | | | | |
| Onset of labour | Not in labour | 696 (62.82%) | 1755 (12.81%) | 6196 (80.05%) | 14,878 (33.47%) | 5525 (72.11%) | 15,581 (32.72%) |
|  | Spontaneous | 327 (29.51%) | 10,715 (78.22%) | 855 (11.05%) | 25,320 (56.95%) | 974 (12.71%) | 26,134 (54.88%) |
|  | Induced | 53 (4.78%) | 591 (4.31%) | 343 (4.43%) | 1292 (2.91%) | 747 (9.75%) | 2182 (4.58%) |
|  | *Missing* | 32 (2.89%) | 637 (4.65%) | 346 (4.47%) | 2968 (6.68%) | 416 (5.43%) | 3725 (7.82%) |
| Mode of delivery | Spontaneous vaginal delivery | 266 (24.0%) | 8987 (65.61%) | 378 (4.88%) | 14,924 (33.57%) | 574 (7.49%) | 16,420 (34.48%) |
|  | Instrumental delivery | 10 (0.90%) | 217 (1.58%) | 57 (0.74%) | 1368 (3.08%) | 89 (1.26%) | 2185 (4.59%) |
| *Caesarean section (CS)* | All CS | 722 (65.16%) | 3813 (27.84%) | 6160 (79.59%) | 23,209 (52.20%) | 5429 (70.86%) | 20,512 (43.07%) |
|  | CS pre-labour | 599 (54.06%) | 1563 (11.41%) | 5210 (67.31%) | 12,125 (27.27%) | 4222 (55.10%) | 10,298 (21.62%) |
|  | CS in labour | 76 (6.86%) | 1993 (14.55%) | 445 (5.75%) | 9193 (20.68%) | 481 (6.28%) | 8189 (17.20%) |
|  | CS post induction | 31 (2.80% | 94 (0.69%) | 264 (3.41%) | 685 (1.54%) | 481 (6.28%) | 879 (1.85%) |
|  | *Missing* | 110 (9.93%) | 681 (4.97%) | 1145 (14.79%) | 4956 (11.15%) | 1570 (20.49%) | 8505 (17.86%) |
| Complications | Prolonged/ preterm rupture of membranes | 96 (8.66%) | 3096 (22.60%) | 248 (3.20%) | 8008 (18.01%) | 281 (3.66%) | 7443 (15.63%) |
|  | Chorio-amnionitis | 40 (3.61%) | 1238 (9.04%) | 54 (0.70%) | 1486 (3.34%) | 18 (0.23%) | 650 (1.36%) |

*Data are summarised as counts (%) for categorical data, mean (standard deviation) for approximately normally distributed continuous variables and median [interquartile range] for skewed continuous variables. IMD = Index of multiple deprivation, SLE = Systemic lupus erythematosus.*

**Table S4.** Survival with and without comorbidity to discharge stratified by gestational age at birth, birthweight centile and HDP status in singleton infants with birthweight centile and comorbidity data (N = 88,285).

*Comorbidity = any of retinopathy of prematurity, severe necrotising enterocolitis or severe brain injury +/- bronchopulmonary dysplasia. BPD = Bronchopulmonary dysplasia. Data for infants born at 23 and 24 weeks’ gestation are merged due to small numbers. Cells with count less than three (0,1 and 2) are suppressed in keeping with latest Office of National Statistics guidance (Disclosure control guidance for birth and death statistics: https://www.ons.gov.uk/methodology/methodologytopicsandstatisticalconcepts/disclosurecontrol/policyonprotectingconfidentialityintablesofbirthanddeathstatistics).*

|  |  | **Birthweight < 10^th^ centile** | | **Birthweight ≥ 10^th^ centile** | |
| --- | --- | --- | --- | --- | --- |
| **Gestational age at birth** | **Survival and comorbidity status** | **HDP** | **No HDP** | **HDP** | **No HDP** |
| 23/24 weeks  N = 4173 | Survived no comorbidity | <3 | 3 (1.6%) | 3 (2.4%) | 145 ( 3.8 %) |
|  | Survived BPD only | 17 ( 25 %) | 43 ( 22.4 %) | 40 ( 31.7 %) | 887 ( 23.4 %) |
|  | Survived + comorbidity +/- BPD | 19 ( 27.9 %) | 50 ( 26 %) | 46 ( 36.5 %) | 1278 ( 33.7 %) |
| 25  N = 3091 | Survived no comorbidity | 6 ( 4.5 %) | 12 ( 6.7 %) | 17 ( 15.2 %) | 345 ( 12.9 %) |
|  | Survived BPD only | 43 ( 32.1 %) | 61 ( 34.3 %) | 47 ( 42 %) | 1004 ( 37.6 %) |
|  | Survived + comorbidity +/- BPD | 41 ( 30.6 %) | 51 ( 28.7 %) | 34 ( 30.4 %) | 856 ( 32.1 %) |
| 26  N = 3915 | Survived no comorbidity | 32 ( 9.6 %) | 40 ( 11.6 %) | 43 ( 22.3 %) | 814 ( 26.7 %) |
|  | Survived BPD only | 130 ( 39.0 %) | 143 ( 41.4 %) | 93 ( 48.2 %) | 1167 ( 38.3 %) |
|  | Survived + comorbidity +/- BPD | 104 ( 31.2 %) | 75 ( 21.7 %) | 41 ( 21.2 %) | 689 ( 22.6 %) |
| 27  N = 4701 | Survived no comorbidity | 98 ( 20.3 %) | 91 ( 16.9 %) | 107 ( 38.1 %) | 1487 ( 43.8 %) |
|  | Survived BPD only | 243 ( 50.3 %) | 260 ( 48.1 %) | 114 ( 40.6 %) | 1101 ( 32.4 %) |
|  | Survived + comorbidity +/- BPD | 77 ( 15.9 %) | 112 ( 20.7 %) | 46 ( 16.4 %) | 575 ( 16.9 %) |
| 28  N = 6110 | Survived no comorbidity | 245 ( 35 %) | 268 ( 32.2 %) | 274 ( 57.7 %) | 2479 ( 60.4 %) |
|  | Survived BPD only | 333 ( 47.5 %) | 360 ( 43.3 %) | 133 ( 28 %) | 941 ( 22.9 %) |
|  | Survived + comorbidity +/- BPD | 85 ( 12.1 %) | 99 ( 11.9 %) | 51 ( 10.7 %) | 457 ( 11.1 %) |
| 29  N = 6829 | Survived no comorbidity | 349 ( 54.4 %) | 424 ( 49.8 %) | 399 ( 69.2 %) | 3553 ( 74.7 %) |
|  | Survived BPD only | 233 ( 36.3 %) | 295 ( 34.7 %) | 134 ( 23.2 %) | 631 ( 13.3 %) |
|  | Survived + comorbidity +/- BPD | 48 ( 7.5 %) | 78 ( 9.2 %) | 38 ( 6.6 %) | 438 ( 9.2 %) |
| 30  N = 8887 | Survived no comorbidity | 530 ( 76.5 %) | 755 ( 70.7 %) | 679 ( 83.7 %) | 5287 ( 83.7 %) |
|  | Survived BPD only | 119 ( 17.2 %) | 225 ( 21.1 %) | 92 ( 11.3 %) | 484 ( 7.7 %) |
|  | Survived + comorbidity +/- BPD | 34 ( 4.9 %) | 60 ( 5.6 %) | 35 ( 4.3 %) | 398 ( 6.3 %) |
| 31  N = 11,517 | Survived no comorbidity | 810 ( 88 %) | 1073 ( 81.4 %) | 955 ( 90.3 %) | 7377 ( 89.7 %) |
|  | Survived BPD only | 72 ( 7.8 %) | 156 ( 11.8 %) | 60 ( 5.7 %) | 398 ( 4.8 %) |
|  | Survived + comorbidity +/- BPD | 31 ( 3.4 %) | 50 ( 3.8 %) | 31 ( 2.9 %) | 318 ( 3.9 %) |
| 32  N = 16,218 | Survived no comorbidity | 1057 ( 97.4 %) | 1768 ( 94.3 %) | 1487 ( 97.4 %) | 11328 ( 96.5 %) |
|  | Survived + comorbidity +/- BPD | 25 ( 2.3 %) | 68 ( 3.6 %) | 31 ( 2 %) | 287 ( 2.4 %) |
| 33  N = 22,844 | Survived no comorbidity | 1284 ( 98.3 %) | 2351 ( 96.7 %) | 1820 ( 98.2 %) | 16887 ( 97.9 %) |
|  | Survived + comorbidity +/- BPD | 18 ( 1.4 %) | 56 ( 2.3 %) | 26 ( 1.4 %) | 269 ( 1.6 %) |

**Table S5.** Survival with and without comorbidity to discharge stratified by gestational age at birth, birthweight centile and HDP status in multi-fetal infants with birthweight centile and comorbidity data (N = 33,412).

*Comorbidity = any of retinopathy of prematurity, severe necrotising enterocolitis or severe brain injury +/- bronchopulmonary dysplasia. BPD = Bronchopulmonary dysplasia. Data for infants born at 23 and 24 weeks’ gestation are merged due to small numbers. Cells with count less than three (0,1 and 2) are suppressed in keeping with latest Office of National Statistics guidance (Disclosure control guidance for birth and death statistics: https://www.ons.gov.uk/methodology/methodologytopicsandstatisticalconcepts/disclosurecontrol/policyonprotectingconfidentialityintablesofbirthanddeathstatistics).*

|  |  | **Birthweight < 10^th^ centile** | | **Birthweight ≥ 10^th^ centile** | |
| --- | --- | --- | --- | --- | --- |
| **Gestational age at birth** | **Survival and comorbidity status** | **HDP** | **No HDP** | **HDP** | **No HDP** |
| 23/24 weeks  N = 1297 | Survived BPD only | < 3 | 31 ( 22.3 %) | 10 ( 40 %) | 226 ( 20.1 %) |
|  | Survived + comorbidity +/- BPD | < 3 | 21 ( 15.1 %) | 9 ( 36 %) | 372 ( 33.2 %) |
|  | Survived no comorbidity | None recorded | 4 ( 2.9 %) | < 3 | 27 ( 2.4 %) |
| 25  N = 956 | Survived BPD only | 4 ( 30.8 %) | 28 ( 26.2 %) | 7 ( 23.3 %) | 270 ( 33.5 %) |
|  | Survived + comorbidity +/- BPD | 5 ( 38.5 %) | 31 ( 29 %) | 16 ( 53.3 %) | 295 ( 36.6 %) |
|  | Survived no comorbidity | None recorded | 6 ( 5.6 %) | 3 ( 10 %) | 63 ( 7.8 %) |
| 26  N = 1155 | Survived no comorbidity | 3 ( 17.6 %) | 17 ( 11.9 %) | 8 ( 21.6 %) | 201 ( 21 %) |
|  | Survived BPD only | 4 ( 23.5 %) | 65 ( 45.5 %) | 16 ( 43.2 %) | 422 ( 44.1 %) |
|  | Survived + comorbidity +/- BPD | 6 ( 35.3 %) | 28 ( 19.6 %) | 9 ( 24.3 %) | 222 ( 23.2 %) |
| 27  N = 1501 | Survived no comorbidity | 7 ( 16.7 %) | 32 ( 15.8 %) | 21 ( 44.7 %) | 478 ( 39.5 %) |
|  | Survived BPD only | 23 ( 54.8 %) | 102 ( 50.5 %) | 16 ( 34 %) | 467 ( 38.6 %) |
|  | Survived + comorbidity +/- BPD | 9 ( 21.4 %) | 34 ( 16.8 %) | 6 ( 12.8 %) | 192 ( 15.9 %) |
| 28  N = 2058 | Survived no comorbidity | 15 ( 27.3 %) | 102 ( 33.8 %) | 55 ( 59.1 %) | 956 ( 59.5 %) |
|  | Survived BPD only | 25 ( 45.5 %) | 127 ( 42.1 %) | 21 ( 22.6 %) | 414 ( 25.7 %) |
|  | Survived + comorbidity +/- BPD | 7 ( 12.7 %) | 39 ( 12.9 %) | 9 ( 9.7 %) | 168 ( 10.4 %) |
| 29  N = 2446 | Survived no comorbidity | 35 ( 52.2 %) | 202 ( 51.9 %) | 87 ( 79.1 %) | 1450 ( 77.1 %) |
|  | Survived BPD only | 24 ( 35.8 %) | 132 ( 33.9 %) | 13 ( 11.8 %) | 270 ( 14.4 %) |
|  | Survived + comorbidity +/- BPD | 6 ( 9 %) | 36 ( 9.3 %) | 9 ( 8.2 %) | 133 ( 7.1 %) |
| 30  N = 3308 | Survived no comorbidity | 57 ( 68.7 %) | 354 ( 65.6 %) | 138 ( 84.1 %) | 2164 ( 85.8 %) |
|  | Survived BPD only | 20 ( 24.1 %) | 147 ( 27.2 %) | 14 ( 8.5 %) | 233 ( 9.2 %) |
|  | Survived + comorbidity +/- BPD | 4 ( 4.8 %) | 28 ( 5.2 %) | 10 ( 6.1 %) | 107 ( 4.2 %) |
| 31  N = 4503 | Survived no comorbidity | 96 ( 86.5 %) | 607 ( 81.4 %) | 258 ( 89.3 %) | 3117 ( 92.9 %) |
|  | Survived BPD only | 12 ( 10.8 %) | 110 ( 14.7 %) | 25 ( 8.7 %) | 136 ( 4.1 %) |
|  | Survived + comorbidity +/- BPD | < 3 | 24 ( 3.2 %) | 5 ( 1.7 %) | 78 ( 2.3 %) |
| 32  N = 7004 | Survived no comorbidity | 232 ( 97.9 %) | 1272 ( 97.7 %) | 527 ( 99.1 %) | 4851 ( 98.3 %) |
|  | Survived + comorbidity +/- BPD | < 3 | 23 ( 1.8 %) | 4 ( 0.8 %) | 62 ( 1.3 %) |
| 33  N = 9184 | Survived no comorbidity | 291 ( 98.6 %) | 1740 ( 98.8 %) | 818 ( 99.4 %) | 6234 ( 98.9 %) |
|  | Survived + comorbidity +/- BPD | 3 ( 1.0 %) | 17 ( 1.0 %) | 4 ( 0.5 %) | 52 ( 0.8 %) |

**Table S6.** Adjusted multivariable logistic regression model for binary outcome survival to discharge in all infants (complete case analysis, N = 95,993).

| **Variable** |  | **Odds ratio (95% Confidence interval)** | **p-value** |
| --- | --- | --- | --- |
| Gestational week at birth  *Reference: 23 weeks’ gestation* | Gestational weeks = 24 | 2.4 (2.12-2.72) | <0.00001 |
|  | Gestational weeks = 25 | 5.33 (4.67-6.08) | <0.00001 |
|  | Gestational weeks = 26 | 8.72 (7.6-10.01) | <0.00001 |
|  | Gestational weeks = 27 | 17.84 (15.35-20.75) | <0.00001 |
|  | Gestational weeks = 28 | 25.09 (21.53-29.24) | <0.00001 |
|  | Gestational weeks = 29 | 57.13 (47.67-68.48) | <0.00001 |
|  | Gestational weeks = 30 | 85.4 (70.59-103.31) | <0.00001 |
|  | Gestational weeks = 31 | 115.71 (95.49-140.22) | <0.00001 |
|  | Gestational weeks = 32 | 191.79 (156.34-235.27) | <0.00001 |
|  | Gestational weeks = 33 | 324.21 (260.39-403.68) | <0.00001 |
| Gestational days at birth, adjusted for gestational weeks  *Reference: +0 days* | Gestational days + 1 | 1.2 (1.06-1.35) | 0.00306 |
|  | Gestational days + 2 | 1.32 (1.17-1.48) | 0.00001 |
|  | Gestational days + 3 | 1.44 (1.27-1.62) | <0.00001 |
|  | Gestational days + 4 | 1.41 (1.26-1.59) | <0.00001 |
|  | Gestational days + 5 | 1.62 (1.44-1.82) | <0.00001 |
|  | Gestational days + 6 | 1.81 (1.6-2.04) | <0.00001 |
| Birthweight centile  *Reference: < 3^rd^ centile* | Birthweight centile 3^rd^ – 9.9^th^ | 2.12 (1.83-2.46) | <0.00001 |
|  | Birthweight centile 10^th^ – 19.9^th^ | 2.87 (2.48-3.31) | <0.00001 |
|  | Birthweight centile 20^th^ -29.9^th^ | 3.06 (2.65-3.53) | <0.00001 |
|  | Birthweight centile 30^th^ -39.9^th^ | 3.35 (2.9-3.87) | <0.00001 |
|  | Birthweight centile 40^th^ -49.9^th^ | 3.52 (3.03-4.08) | <0.00001 |
|  | Birthweight centile 50^th^ -59.9^th^ | 3.69 (3.16-4.3) | <0.00001 |
|  | Birthweight centile 60^th^ -69.9^th^ | 3.67 (3.11-4.33) | <0.00001 |
|  | Birthweight centile 70^th^ -79.9^th^ | 3.59 (3-4.31) | <0.00001 |
|  | Birthweight centile 80^th^ -89.9^th^ | 3.06 (2.49-3.76) | <0.00001 |
|  | Birthweight centile 90^th^ + | 1.38 (1.12-1.7) | 0.00233 |
| Maternal antenatal steroid course  *Reference: None given* | Complete course (2 or more doses) | 2.4 (2.13-2.7) | <0.00001 |
|  | Incomplete course (1 dose) | 1.97 (1.73-2.24) | <0.00001 |
| Mode of delivery  *Reference: Emergency caesarean section* | Spontaneous vaginal delivery | 1.20 (1.11-1.3) | 0.00001 |
|  | Instrumental vaginal delivery | 1.26 (0.98-1.62) | 0.06973 |
|  | Elective caesarean section | 1.19 (0.74-1.89) | 0.47331 |
| Hypertensive disorder of pregnancy |  | 1.47 (1.3-1.66) | <0.00001 |
| Biological sex | Female fetus | 1.28 (1.2-1.37) | <0.00001 |
| Multifetal pregnancy |  | 1.01 (0.94-1.1) | 0.73198 |

**Table S7.** Comparison of ONS livebirth and study cohort maternal ethnicity data for Asian, Black and White ethnic groups 2014-2020.

| **Ethnic group** | **ONS 2014-2020 livebirths < 34 weeks**  **n = 102,210** | **Study cohort 2014-2020 < 34 weeks**  **N = 122,288** |
| --- | --- | --- |
| Asian | 9,561 (9.4%) | 9,897 (10.5%) |
| Black | 6,402 (6.3%) | 6,042 (6.4%) |
| White | 69,746 (68.2%) | 57,446 (61.0%) |

**Figures**

**Figure S1:** Flow diagram of data cleaning steps.


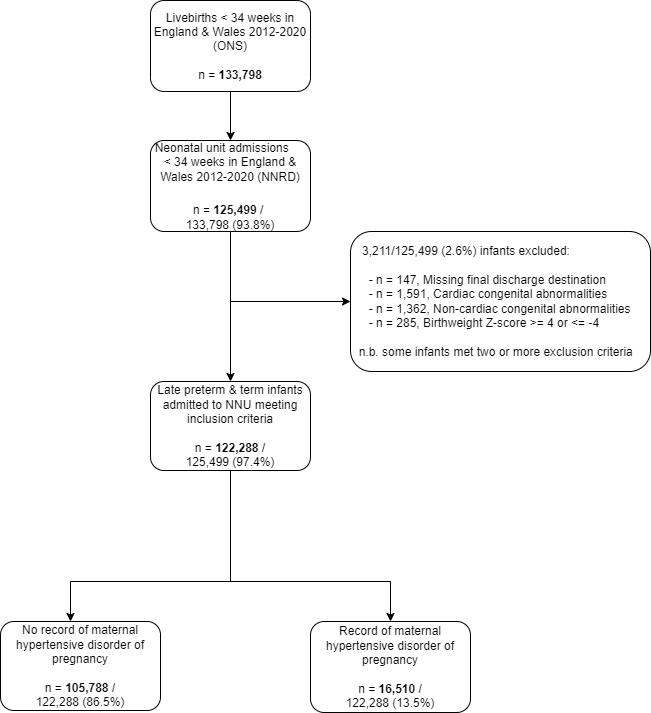


**Figure S2:** Percentage of live-born babies < 34 weeks’ gestation without congenital abnormalities admitted to Neonatal Units in England and Wales with a maternal record of hypertensive disorder of pregnancy (HDP) by (A) gestational age at delivery and (B) year of birth. Bars are filled according to onset of labour.

**A**


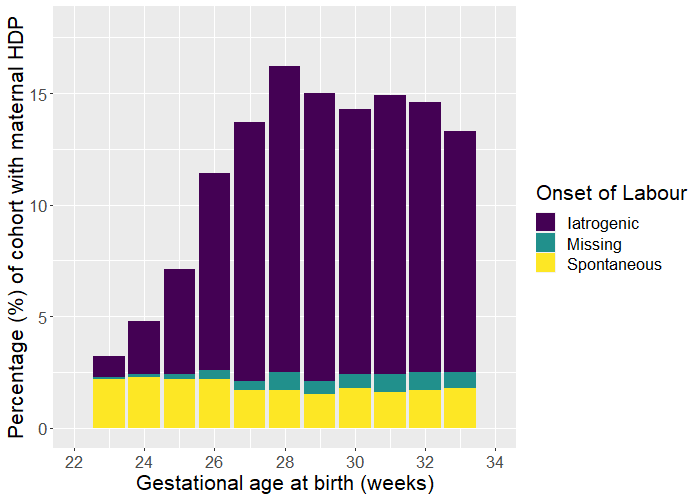


**B**

**
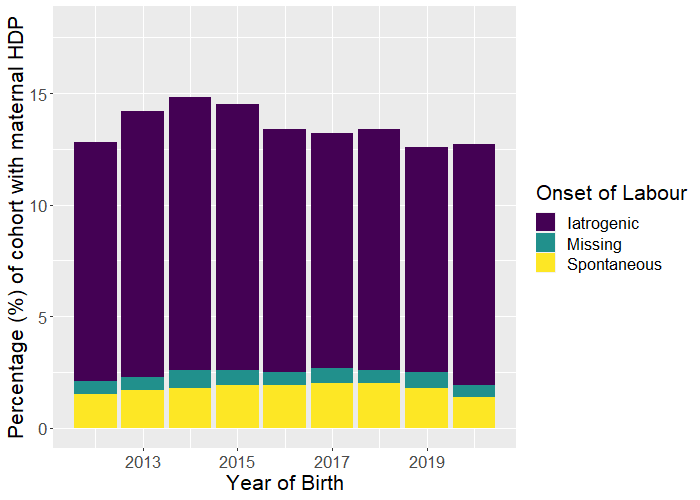
**

**Figure S3:** Cumulative frequency chart of gestational age at delivery stratified by maternal ethnic groups (Asian, Black and White displayed) in HDP exposed (A) and all (B) infants.

**A. HDP exposed infants born to mothers of Asian, Black or White ethnic backgrounds (N = 13,610)**

**
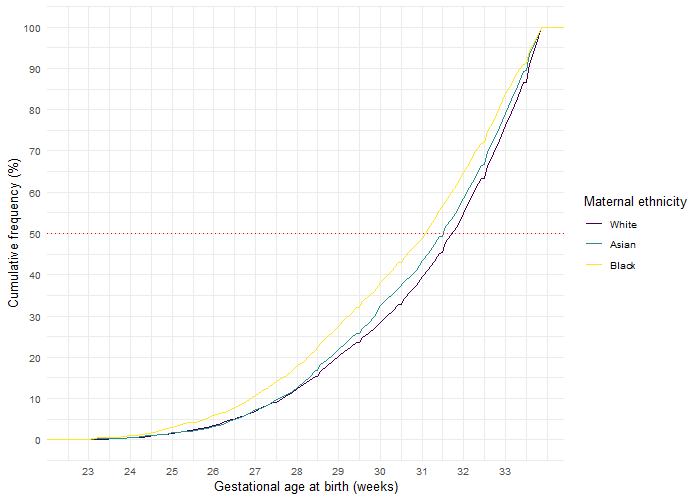
**

**B. All infants born to mothers of Asian, Black or White ethnic backgrounds (N = 98,809)**


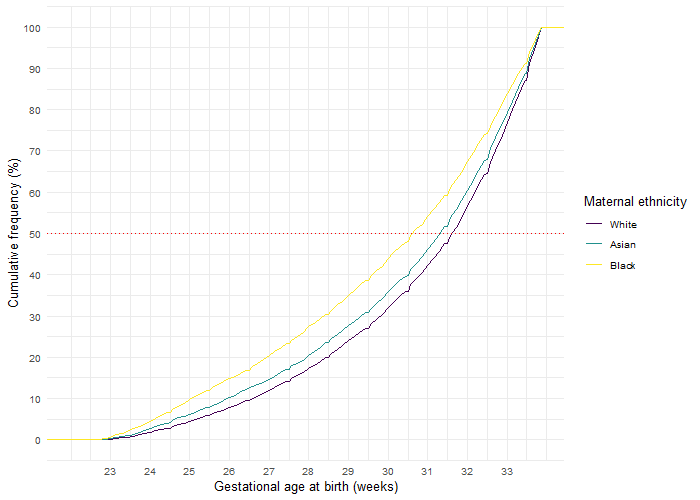

Supplement: Supplementary file 1 — Data S1. [file BJO-132-306-s002.docx]
